# Artificial Intelligence in Rehabilitation Targeting the Participation of Children and Youth With Disabilities: Scoping Review

**DOI:** 10.2196/25745

**Published:** 2021-11-04

**Authors:** Vera C Kaelin, Mina Valizadeh, Zurisadai Salgado, Natalie Parde, Mary A Khetani

**Affiliations:** 1 Rehabilitation Sciences College of Applied Health Sciences University of Illinois at Chicago Chicago, IL United States; 2 Children's Participation in Environment Research Lab University of Illinois at Chicago Chicago, IL United States; 3 Computer Science College of Engineering University of Illinois at Chicago Chicago, IL United States; 4 Natural Language Processing Laboratory University of Illinois at Chicago Chicago, IL United States; 5 Occupational Therapy College of Applied Health Sciences University of Illinois at Chicago Chicago, IL United States; 6 CanChild Centre for Childhood Disability Research McMaster University Hamilton, ON Canada

**Keywords:** health care, pediatric rehabilitation, technology, young persons, robotics, human-machine interaction, personalization, customization, goal-setting, natural language processing, machine learning

## Abstract

**Background:**

In the last decade, there has been a rapid increase in research on the use of artificial intelligence (AI) to improve child and youth participation in daily life activities, which is a key rehabilitation outcome. However, existing reviews place variable focus on participation, are narrow in scope, and are restricted to select diagnoses, hindering interpretability regarding the existing scope of AI applications that target the participation of children and youth in a pediatric rehabilitation setting.

**Objective:**

The aim of this scoping review is to examine how AI is integrated into pediatric rehabilitation interventions targeting the participation of children and youth with disabilities or other diagnosed health conditions in valued activities.

**Methods:**

We conducted a comprehensive literature search using established Applied Health Sciences and Computer Science databases. Two independent researchers screened and selected the studies based on a systematic procedure. Inclusion criteria were as follows: participation was an explicit study aim or outcome or the targeted focus of the AI application; AI was applied as part of the provided and tested intervention; children or youth with a disability or other diagnosed health conditions were the focus of either the study or AI application or both; and the study was published in English. Data were mapped according to the types of AI, the mode of delivery, the type of personalization, and whether the intervention addressed individual goal-setting.

**Results:**

The literature search identified 3029 documents, of which 94 met the inclusion criteria. Most of the included studies used multiple applications of AI with the highest prevalence of robotics (72/94, 77%) and human-machine interaction (51/94, 54%). Regarding mode of delivery, most of the included studies described an intervention delivered in-person (84/94, 89%), and only 11% (10/94) were delivered remotely. Most interventions were tailored to groups of individuals (93/94, 99%). Only 1% (1/94) of interventions was tailored to patients’ individually reported participation needs, and only one intervention (1/94, 1%) described individual goal-setting as part of their therapy process or intervention planning.

**Conclusions:**

There is an increasing amount of research on interventions using AI to target the participation of children and youth with disabilities or other diagnosed health conditions, supporting the potential of using AI in pediatric rehabilitation. On the basis of our results, 3 major gaps for further research and development were identified: a lack of remotely delivered participation-focused interventions using AI; a lack of individual goal-setting integrated in interventions; and a lack of interventions tailored to individually reported participation needs of children, youth, or families.

## Introduction

### Background

Technology-based interventions are of increased importance in pediatric rehabilitation and can be useful to rehabilitation practitioners when delivering family-centered and function-focused interventions to service-eligible children, youth, and families [1]. In addition, technology-based rehabilitation tools can be useful to organizations that have electronic data capture systems to monitor trends in rehabilitation service use and outcomes for quality improvement [2,3]. For both individuals and organizations, the COVID-19 pandemic has heightened the demand for technological solutions to remotely deliver and monitor rehabilitation services [4].

One way to provide technology-based pediatric rehabilitation is by applying artificial intelligence (AI), which is a priority of the National Institutes of Health, as reflected in their Rehabilitation Research Plan [5,6]. According to Russell and Norvig [7], AI is concerned with designing and building systems that think like humans, act like humans, think rationally, and act rationally. It encompasses different subfields such as natural language processing (NLP), robotics, or human augmentics [7]. The application of AI in pediatric rehabilitation has the potential to simplify steps in the therapeutic process and possibly decrease provider and patient burden as well as afford providers to customize their rehabilitation services.

Rehabilitation includes a broad range of highly variable interventions that are challenging to define owing to their complexity [8-10]. One important way to classify rehabilitation intervention is through its targeted outcome [9]. In the last decade, there has been a rapid increase in research on the use of AI to improve key pediatric rehabilitation outcomes, including body functions, activity performance, and the full participation of children and youth with disabilities in valued activities [11-13]. For children and youth, participation in home, school, and community activities has been defined by the World Health Organization as “involvement in life situations*”* [14] and was further conceptualized by Imms et al [15] as attendance and involvement in activities, which is related to but distinct from their activity competencies, environment or context, and their preferences or sense of self [15]. Given the unmet participation need among children or youth with disabilities and other diagnosed health conditions, beginning in early childhood and across settings [16-19], there is a growing number of participation-focused intervention studies [20], including interventions that integrate AI to target the participation of young persons receiving pediatric rehabilitation.

A recent systematic literature review on the effect of participation-focused pediatric rehabilitation identified 2257 records through a database search, indicating the high relevance of participation as an outcome in pediatric rehabilitation interventions [20]. However, this review does not focus on AI use. Literature reviews focusing on AI indicate that the use of AI in the form of information and communication technology or robots may improve children’s engagement in play, stimulate school performance [13], and promote social interactions [11,12]. However, these reviews place variable focus on participation [11-13], are narrow in scope (eg, focus on participation in play only) [13], and are restricted to select diagnoses (eg, physical disability) [13]. These limitations hinder our understanding of the existing scope of AI applications in pediatric rehabilitation that target participation in daily life activities.

### Objectives

To better understand the current scope of AI applications within pediatric rehabilitation and to identify gaps for future research, there is a critical need to summarize existing evidence on the use of AI across interventions targeting child and youth participation in activities. The purpose of this scoping review is to examine how AI is integrated into pediatric rehabilitation interventions targeting the participation of children and youth with disabilities or other diagnosed health conditions in valued activities.

Our paper’s contributions are as follows:

1. An overview of the scope of literature focusing on AI targeting participation as a primary pediatric rehabilitation outcome and top priority from the perspective of families.
2. A summary of the types of AI and personalization used in the interventions over a time span of more than 20 years.
3. Identification of research gaps based on the found and summarized literature with a focus on AI targeting the participation of children and youth with disabilities or other diagnosed health conditions.

## Methods

### Design

Scoping reviews are commonly used to provide an overview of existing evidence in a certain field and identify gaps for future research [21,22]. The increasing number of publications on the use of AI in participation-focused pediatric rehabilitation indicates an emerging field for the advancement of rehabilitation research and therefore justifies the need to conduct this scoping review [21]. The protocol for this scoping review was registered in the Open Science Framework [23].

### Search Strategy

**Figure 1:**
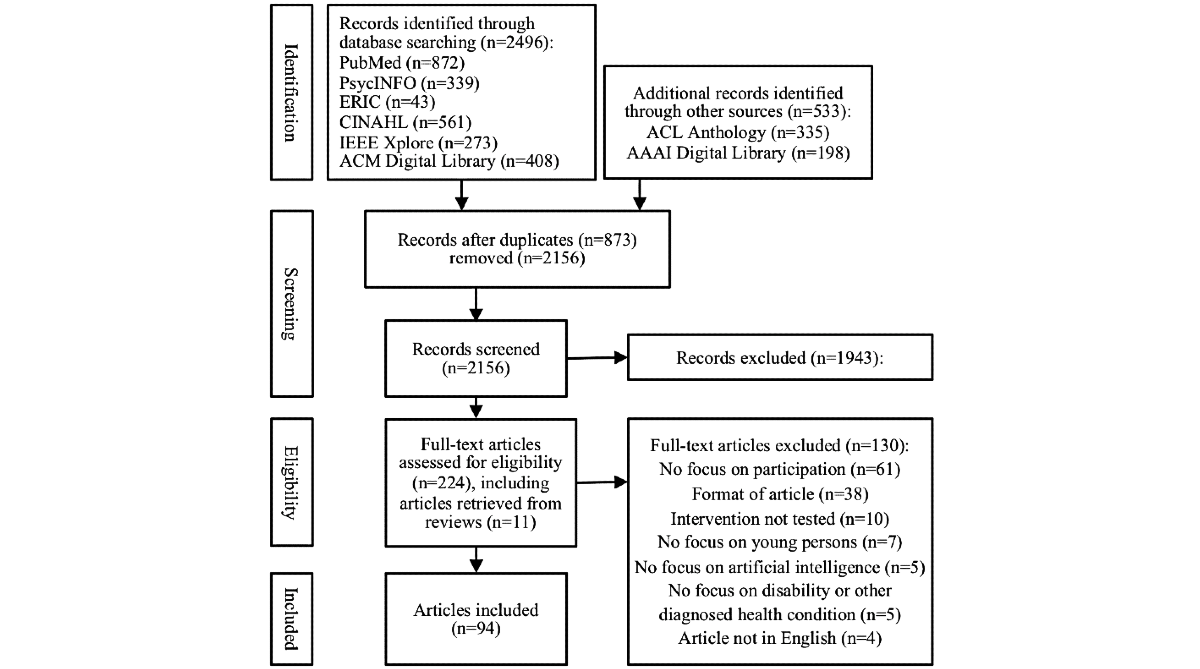
Study selection process.

The first author of this review (VCK) conducted a systematic literature search in well-established databases in the fields of Applied Health Sciences and Computer Science (PubMed, PsycINFO, ERIC, CINAHL, IEEE Xplore, and ACM Digital Library) for documents published before February 2021. No other publication data limit or search limitations were applied to the search. We solicited support from a health sciences librarian to develop subject headings for each database with available thesaurus (ie, PubMed, PsycINFO, ERIC, and CINAHL) and keywords for *artificial intelligence*, *participation*, *health care*, *disability*, and *young persons* (Textbox 1) [24,25]. These were applied using truncations and Boolean terms, resulting in 2496 documents (Figure 1). The full search strategy is presented in Multimedia Appendix 1. After consultation with an AI expert (NP), additional searches were performed in ACL Anthology and AAAI Digital Library, using the same keywords from the database searches. This led to an additional 533 documents.

**Search strategy.**

**Main search term and additional search terms for abstract and title search**

- artificial intelligence
  affective computing, algorithms, chatbot, cognitive computing, computer vision, constraint optimization, constraint satisfaction, data mining, data processing, deep learning, expert systems, feature extraction, fuzzy logic, game theory, human computation, image analysis, inductive logic programming, knowbot*, knowledge bases, knowledge-based agent, knowledge engineering, knowledge representation, machine learning, natural language processing, neural networks, pattern recognition, predictive model, reinforcement learning, robot*, semantic networks, semi-supervised learning, supervised learning, text analysis, unsupervised learning, virtual agent, virtual reality
- participation
  attendance, engag*, inclus*, involvement
- health care
  health care, healthcare, rehabilitation, therap*
- disability
  disab*, handicap*, impair*, special needs, special need
- young persons
  adolesc*, caregiv*, child*, family, families, infant*, paediatric*, parent*, pediatric*, student*, teen*, toddler*, young adult, young adults, youth*

### Screening and Selection Process

After removal of duplicates, 2 independent coders (VCK and MV) applied inclusion and exclusion criteria to the title and abstracts of the remaining documents. Documents indicating potential fit based on their abstract or title underwent full-text reading and were coded based on the same inclusion and exclusion criteria. First, 2 independent authors with expertise in participation-focused pediatric rehabilitation (VCK and ZS) coded the same documents until at least 80% agreement was reached [26]. Discrepancies and coding uncertainties were resolved through discussion. The remaining documents were screened separately (VCK and ZS), whereas 20% of randomly selected documents underwent double screening (ie, 10%: VCK and ZS; 10% including an external reviewer with expertise in participation-focused pediatric rehabilitation: VCK and Kyle A Truevillian) [26]. Doubts regarding document inclusion were discussed with a third reviewer (VCK, ZS, and Kyle A Truevillian). Second, documents indicating fit were further screened by an additional author (MV) with a specific focus on AI. Disagreements were resolved through discussion (VCK, ZS, MV, and Kyle A Truevillian) and with the help of additional key informants (MAK and NP).

Documents were included if (1) participation was an explicit study aim or outcome, or the targeted focus of the AI application; (2) AI was applied as part of the provided and tested intervention; (3) children or youth with a disability or other diagnosed health condition were the focus of either the study, AI application or both; and (4) the study was published in English. To ensure the inclusion of a broad scope of studies, no operational definition of participation was used when applying the selection criteria. Studies or AI interventions focused on participation; inclusion; engagement; playfulness; access to, or attendance in life situations, settings or activities; social interaction; or social engagement were considered participation and were therefore included in this review. Documents were excluded if (1) participation in daily activities was not the focus of the study (eg, focus was on skill development); (2) there was no use of AI for the described intervention (eg, the term *algorithm* was used in a noncomputer science way); (3) interventions using AI were not tested with either children, youth or both; (4) there was no focus on disability or other diagnosed health condition; (5) studies focused on adults (mean age >24 years [27]); (6) the study was published in languages other than English; or (7) documents were textbooks or textbook reviews, literature reviews, study protocols, conference or workshop programs, or only abstracts without additional information. To prevent missing relevant studies, each reference list of the excluded literature reviews was screened.

### Data Extraction and Analysis

The data extraction template using Microsoft Excel was trialed with 5 studies by the first author and discussed with the author team to ensure the clarity and relevance of the included categories for data extraction [28]. Data were then extracted for all included studies based on the following categories: authors, year, title, sample size, child and youth age, child and youth race and ethnicity, family socioeconomic status, parental education, the types of AI applied in the intervention, the intervention’s type of personalization, whether the primary method for intervention delivery was in-person or remote, and whether goal-setting was addressed as part of the intervention.

The mapping of included studies to one or multiple types of AI was guided by CSRankings [29] and the AAAI keywords taxonomy [30], 2 commonly used ranking systems and taxonomies for AI. The types of AI in this study include robotics, NLP, human computation and crowdsourcing, computer vision, knowledge representation and reasoning, machine learning (ML), human-machine interaction (HMI), cognitive modeling, constraint satisfaction and optimization, game theory, planning and routing and scheduling, and visualization and virtual reality (VR) [7]. As robotic devices are increasingly used in rehabilitation [31], studies that used robotics were further classified according to whether they focused on robot mechanics or on the system of use.

A framework developed by Fan et al [32] guided the mapping of the intervention’s type of personalization according to 2 dimensions (ie, categorical vs individuated personalization; implicit vs explicit personalization). Categorical personalization targets a category of individuals, such as a diagnostic group or single-child families [32]. For this review, these were, for example, devices that are designed to include features that meet the common needs of children with autism spectrum disorder (ASD). Individuated personalization targets specific individuals [32], in the case of this review, individually perceived and reported participation needs. Implicit personalization is system-initiated, meaning it is automatically done by the system, whereas explicit personalization is user-initiated, meaning users manually guide the system on the preferred adaptation [32].

Interventions were mapped as in-person when the intervention was delivered face-to-face with a researcher or rehabilitation professional. Interventions were considered as remotely delivered when they were conducted in the child’s natural environment and without a researcher or rehabilitation professional.

Mapping of included studies with regard to whether goal-setting was addressed as part of the described rehabilitation services was guided by the goal-setting and action-planning practice framework for rehabilitation settings [33]. Studies were mapped to address goal-setting if the described rehabilitation services included goal negotiation (ie, where the patient is at and where the patient would like to get to) or goal-setting (ie, what the patient would like to achieve) [33].

Charted data were summarized using descriptive statistics (ie, frequency counts and percentages) to provide an overview of the available evidence on how AI is used to support participation among children and youth with disabilities or other diagnosed health conditions.

## Results

### Overview of Found and Included Research

The literature search revealed 3029 documents with 873 duplicates (ie, documents appeared multiple times), resulting in 2156 documents entering the 2-fold screening process to assess their eligibility based on the inclusion and exclusion criteria (Figure 1). The first screening phase included titles and abstracts and led to 213 included and 1943 excluded documents, as well as 11 additional studies found when screening the reference list of excluded literature reviews. The Cohen κ for interrater agreement was 0.67, indicating a substantial agreement [34]. This estimate did not include the numerous conference programs (n=450) found through AAAI Digital Library and ACL Anthology, for which determining exclusion was trivial, resulting in a more conservative Cohen κ value.

The second screening phase included a full-text review of the 224 included documents from the first screening phase, resulting in 94 included studies for this scoping review. Of the 130 excluded documents, 61 (46.9%) lacked focus on participation, 38 (29.2%) were excluded because of their format (ie, textbook or textbook review, study protocol, literature review, or only abstract), 10 (7.7%) did not test the intervention, and 7 (5.4%) addressed an adult population; 5 (3.8%) did not use AI in the intervention, 5 (3.8%) did not focus on people with disability or other diagnosed health conditions, and 4 (3.1%) were not written in English (Figure 1).

### Type of Included Research

The 94 included studies were published between 2000 and 2021, with a higher proportion published after 2010 (76/94, 81%; Multimedia Appendix 2 [35-128]). All studies included AI as part of their intervention and targeted children or youth participation, as described in their research aims, outcomes, or as their focus of the tested AI application.

As for sample characteristics, the described interventions were evaluated on sample sizes ranging from 1 to 120 children and youth with an average of 14 children or youth. Of the included studies that reported on gender identity, 76% (51/67) had a higher proportion of boys represented in their sample. A total of 92% (86/94) of the included studies did not report on the socioeconomic background of the family, parental education, or child or youth race or ethnicity. In total, 15% (14/94) of studies sampled caregivers, teachers, peers, other school staff, or combinations thereof, in addition to children or youth when evaluating the intervention. Included interventions were developed or tested for children or youth with a broad range of diagnoses, with ASD being the most prevalent (43/94, 46%), followed by cerebral palsy (CP; 18/94, 19%).

### Types of AI Intervention, Mode of Intervention Delivery, and Type of Personalization

Most of the 94 included studies used robotics as the type of AI intervention to target participation among children and youth with disabilities or other diagnosed health conditions (72/94, 77%) [35-106], followed by HMI (51/94, 54%) [35, 37-44, 47, 50-55, 58, 65, 66, 69, 71, 72, 75, 78, 80, 82, 85-89, 91-98, 100, 101, 103-111], visualization and VR (19/94, 20%) [53,54,72,79,107,108,112-124], NLP (18/94, 19%) [36,47,52,64,71,78,79,82,91,101,103-105,120,125-128], ML (11/94, 12%), computer vision (10/94, 11%) [40,41,64,67,69,107,110,120,125,126,128], and constraint satisfaction and optimization (1/94, 1%; Table 1) [110]. Of the 72 studies on robotics, 63 (88%) studies focused on the system of use [35-39, 41-53, 55, 57, 58, 60-63, 65, 66, 68-74, 76-78, 80-101, 103-106] and 9 (13%) focused on robot mechanics [40,54,56,59,64,67,75,79,102].

**Table 1:** Delivery of participation-focused rehabilitation interventions that include artificial intelligence (AI).

Type of AI	Personalization	Mode of delivery	Addresses individual goal-setting
Robotics: 72 [35-106]	Implicit + individuated: 0 Implicit + categorical: 37 [35, 36, 38, 43-46, 48-50, 52, 55-57, 60, 64-66, 70, 74-77, 79, 81-84, 87, 89-91, 97, 101, 104-106] Explicit + individuated: 0 Explicit + categorical: 35 [37, 39-42, 47, 51, 53, 54, 58, 59, 61-63, 67-69, 71-73, 78, 80, 85, 86, 88, 92-96, 98-100, 102, 103]	In-person: 67 [35-50,52-84,86-91,93,94,96-99,101-106] Remote: 5 [51,85,92,95,100]	0
Human-machine interaction: 51 [35, 37-44, 47, 50-55, 57, 58, 65, 66, 69, 71, 72, 75, 78, 80, 82, 85-89, 91-98, 100, 101, 103-111]	Implicit + individuated: 1 [110] Implicit + categorical: 21 [35, 38, 43, 44, 50, 52, 55, 57, 65, 66, 75, 82, 87, 89, 91, 97, 101, 104-107] Explicit + individuated: 0 Explicit + categorical: 29 [37, 39-42, 47, 51, 53, 54, 58, 69, 71, 72, 78, 80, 85, 86, 88, 92-96, 98, 100, 103, 108, 109, 111]	In-person: 44 [35, 37-44, 47, 50, 52-55, 57, 58, 65, 66, 69, 71, 72, 75, 78, 80, 82, 86-89, 91, 93, 94, 96-98, 101, 103-107, 109, 111] Remote: 7 [51,85,92,95,100,108,110]	0
Visualization and virtual reality: 19 [53,54,72,79,107,108,112-124]	Implicit + individuated: 0 Implicit + categorical: 5 [79,107,114,117,120] Explicit + individuated: 0 Explicit + categorical: 14 [53,54,72,108,112,113,115,116,118,119,121-124]	In-person: 18 [53,54,72,79,107,112-124] Remote: 1 [108]	1 [112]
Natural language processing: 18 [36, 47, 52, 64, 71, 78, 79, 82, 91, 101, 103-105, 120, 125-128]	Implicit + individuated: 0 Implicit + categorical: 14 [36,52,64,79,82,91,101,104,105,120,125-128] Explicit + individuated: 0 Explicit + categorical: 4 [47,71,78,103]	In-person: 15 [36,47,52,64,71,78,79,82,91,101,103-105,120,127] Remote: 3 [125,126,128]	0
Machine learning: 11 [40,41,64,67,69,107,110,120,125,126,128]	Implicit + individuated: 1 [110] Implicit + categorical: 6 [64,107,120,125,126,128] Explicit + individuated: 0 Explicit + categorical: 4 [40,41,67,69]	In-person: 7 [40,41,64,67,69,107,120] Remote: 4 [110,125,126,128]	0
Computer vision: 10 [35,39,58,63,65,69,75,112,120,127]	Implicit + individuated: 0 Implicit + categorical: 5 [35,65,75,120,127] Explicit + individuated: 0 Explicit + categorical: 5 [39,58,63,69,112]	In-person: 10 [35,39,58,63,65,69,75,112,120,127] Remote: 0	1 [112]
Constraint satisfaction and optimization: 1 [110]	Implicit + individuated: 1 [110] Implicit + categorical: 0 Explicit + individuated: 0 Explicit + categorical: 0	In-person: 0 Remote: 1 [110]	0
Human computation and crowdsourcing: 0	N/A^a^	N/A	N/A
Planning, routing, and scheduling: 0	N/A	N/A	N/A
Cognitive modeling: 0	N/A	N/A	N/A
Game theory: 0	N/A	N/A	N/A

^a^N/A: not applicable.

Most of the included studies described interventions using multiple applications of AI (60/94, 64%), such as robotics with HMI [35-44, 47, 50-55, 57, 58, 63-67, 69, 71, 72, 75, 78-80, 82, 85-89, 91-98, 100, 101, 103-108, 112, 120, 125-128], or ML with NLP and constraint satisfaction and optimization [110]. Across these studies, robotics was most often integrated into interventions that employed multiple applications of AI. Examples of multiple AI interventions that include robotics are humanoid or nonhumanoid devices to facilitate interaction or play of children with disabilities by directing the robot head toward a target or rocking its body from left to right to express emotions such as excitement [39].

Out of the included 94 studies, 22 (23%) studies used forms of AI other than robotics [107-128]. Of these, 15 included visualization and VR applications, such as an immersive virtual learning program [107,108,112-124]; 8% (7/94) of interventions included neither robotics nor visualization and VR [109-111,125-128]. Examples of such interventions are a framework for speech-to-sign language translation for children with hearing impairments [127] and the design of a virtual space for hospitalized children to meet with their peers [108].

As for mode of delivery, most of the included studies described an intervention delivered in-person (84/94, 89%) [35-50,52-84,86-91,93,94,96-99,101-107,109,111-124,127], mainly using a one-on-one approach. A total of 11% (10/94) of included studies evaluated an AI intervention that was delivered remotely [51,85,92,95,100,108,110,125,126,128].

Most AI interventions were tailored to a category of individuals (ie, categorical personalization) such as by a diagnostic group (93/94, 99%) [35-109,111-128], using implicit (ie, automatically personalized: 45/94, 48%) [35, 36, 38, 43-46, 48-50, 52, 55-57, 60, 64-66, 70, 74-77, 79, 81-84, 87, 89-91, 97, 101, 104-107, 114, 117, 120, 125-128] or explicit (ie, manually personalized: 48/94, 51%) approaches [37, 39-42, 47, 51, 53, 54, 58, 59, 61-63, 67-69, 71-73, 78, 80, 85, 86, 88, 92-96, 98-100, 102, 103, 108, 109, 111-113, 115, 116, 118, 119, 121-124]. For example, Yee et al [35] designed a robotic platform for children with ASD by tailoring it to the needs typically described by this diagnostic group. In contrast, only 1% (1/94) of the included studies described an intervention that was tailored to the individually reported and unique needs of the child or youth with a disability or other diagnosed health condition (ie, individuated personalization) [110]. It included the use of a recommender algorithm, integrating information about the location of different physical and virtual learning resources, their purposes, modality, as well as the individual’s class schedules, university rooms, and navigation system to suggest suitable and uniquely tailored options for access and navigation to the appropriate location [110]. In addition, 1% (1/94) of the included interventions described individual goal-setting as part of their therapy process or intervention planning [112]. In this intervention, the Canadian Occupational Performance Measure was used for individual goal-setting and a video game–based task-oriented activity training was performed according to the defined patient goal [112].

## Discussion

### Principal Findings

This study summarizes 2 decades of evidence on the use of AI across interventions targeting the participation of children and youth with disabilities or other diagnosed health conditions, extending knowledge on the breadth of using AI in pediatric rehabilitation. There is an increased interest in AI applications for customizing pediatric rehabilitation services to individual child and family reported needs and reducing provider burden. The results of this review suggest that AI applications designed for children of diverse ages and diagnoses tend to emphasize robotics (alone or in combination with other forms of AI), in-person delivery, and targeted groups of children using implicit and explicit personalization approaches. Each finding is further discussed to identify knowledge gaps that warrant future research.

Most of the studied robotic devices are not commercially available and were used during on-site therapy sessions to *train* a child or youth to participate in a specific activity, with an expected transfer or carryover of that gain into the child or youth’s natural environment of home, school, or community. This expectation has been challenged in previous participation literature, emphasizing the importance of environments for shaping a young person’s participation in daily activities [129-132]. The mediating role of the environment and context for child and youth participation has also been supported in research examining the effect of participation-focused interventions [133,134].

Interestingly, most of the found interventions were delivered in-person, despite the potential for leveraging technology to deliver rehabilitation interventions remotely. This result is in line with a previously conducted survey, which indicated that only 8% of Americans used telemedicine in 2019 [135]. Alternatively, our results might also be due to the high prevalence of interventions using robotics, often requiring the presence of trained operators and specialized equipment on the therapy site [48,52,84]. Remotely delivered interventions using robotics deploy robots in classrooms to enable virtual inclusion of home-bound children [51,85,92,95,100]. The remaining remotely delivered interventions commonly apply ML and NLP, potentially indicating the suitability of ML and NLP for use in remote pediatric rehabilitation interventions using AI. ML and NLP have been used in a range of health interventions to promote behavioral changes, such as physical activity and healthy diet, including goal-setting [136,137]. Given the existing evidence on the use of AI for goal-setting in other health care domains [136,137] and the importance of gaining efficiency in enacting the complex process of goal-setting in pediatric rehabilitation [138,139], the lack of attention to goal-setting in this review indicates a clear knowledge gap warranting future research. Emerging electronic participation–focused interventions such as the Participation and Environment Measure–Plus [140-143] with individual goal-setting as an integral part of their intervention might benefit from exploring the use of AI to fill this knowledge gap.

Most of the identified AI applications were tailored to the needs of groups of individuals, with only 1% (1/94) being tailored to the individually reported participation needs of children and youth with disabilities or other diagnosed health conditions. When comparing this result with the use of AI in fields outside of health care, it is surprising. For example, in marketing, AI has revolutionized common advertisement practices by tailoring advertisements to the reported needs and preferences of clients. This discrepancy between fields might be due to stricter protection of health information; however, there is an increase in similar advancements using data collected from patients in formal (eg, electronic health records) or informal (eg, patient dialog) settings, such as for diagnosing and decision-making [144-148]. Similar approaches might also be possible and beneficial within pediatric rehabilitation, using existing patient data to predict tailored participation–focused interventions. A recent systematic literature review on the effects of participation-focused interventions recommends focusing on individually tailored interventions to support the participation of children and youth with disabilities [20]. One way to tailor rehabilitation interventions to the reported needs of patients involves the patient’s goals. In rehabilitation, goal-setting has become an integral part of the therapy process across professions, including pediatric participation–focused interventions [133,134]. Previous research has shown that caregivers can be guided to create participation-focused goals on the web [149]. Including goal-setting in AI-supported pediatric rehabilitation interventions might be an important first step to enable tailoring interventions to the participation needs of children and youth with disabilities or other diagnosed health conditions.

Despite the high prevalence of included studies testing or designing interventions for children and youth with ASD or cerebral palsy, a diverse sample in terms of diagnoses was represented in this scoping review, indicating relevance for the use of AI applications across diagnoses. In contrast, only 9% (8/94) of studies reported on child or youth race or ethnicity, family socioeconomic status, parental education, or family income, despite evidence indicating its influence on child and youth participation [150-152]. Future research should capture child and youth race and ethnicity as well as indicators of socioeconomic family status to describe the diversity of their study sample [153].

### Limitations

An effort was made to conduct a comprehensive review of the literature pertaining to the use of AI to target children and youth participation. However, the results of this scoping review should be interpreted in light of some limitations. Despite the relatively high number of included studies, we may have missed some relevant documents. Three primary examples include (1) if an intervention using AI was not identified as such during the screening of titles and abstracts, the document was likely excluded from the search or selection process; (2) screening of reference lists was undertaken for review articles versus all included studies; and (3) documents published in languages other than English were excluded. In addition, the included studies were not screened based on their definition of participation, potentially leading to conceptual inconsistency, as has been shown in a systematic review of participation-focused interventions for children with disabilities [20]. Variability in the conceptualization of participation can limit the interpretation and comparison of results across studies [20,154] to identify knowledge gaps specific to participation-focused rehabilitation interventions. Future research should map studies using AI to contemporary frameworks of the participation concept to ensure the interpretability of results across studies.

### Conclusions

There is an increasing amount of research on interventions using AI to target the participation of children and youth with disabilities or other diagnosed health conditions, supporting the potential of using AI in pediatric rehabilitation. Overall, most interventions used multiple AI applications, including robotics and HMI. Other types of AI, such as ML or NLP, were less prevalent but showed potential benefits in participation-focused intervention. On the basis of our results, 3 major gaps were identified, warranting the need for future research and development: (1) a lack of remotely provided participation-focused interventions using AI; (2) a lack of individual goal-setting integrated in interventions using AI; and (3) a lack of interventions using AI tailored to individually reported participation needs of children, youth, or families.

In addition, future research should consistently report on the socioeconomic background of the family, parental education, or race and ethnicity to describe the diversity of their study sample.

## Appendix

Multimedia Appendix 1 Search history in the included databases.

Multimedia Appendix 2 Included studies.

## Abbreviations

AI: artificial intelligence
ASD: autism spectrum disorder
HMI: human-machine interaction
ML: machine learning
NLP: natural language processing
VR: virtual reality
