# Supplementary material for: Artificial Intelligence in Rehabilitation Targeting the Participation of Children and Youth With Disabilities: Scoping Review
Source: J Med Internet Res. 2021 Nov 4;23(11):e25745. doi: 10.2196/25745 (PMC8603165; doi:10.2196/25745)
Supplement: Multimedia Appendix 1 [file jmir_v23i11e25745_app1.pdf]

Review

Appendix A. Search history in the included databases.

Database	Search terms
Pubmed	("algorithms"[mesh] OR "data mining"[mesh] OR "artificial intelligence"[tiab] OR "machine learning"[tiab] OR "natural language processing"[tiab] OR "data mining"[tiab] OR "algorithm*"[tiab] OR "fuzzy logic"[tiab] OR "knowledge bases"[tiab] OR "neural networks"[tiab] OR "deep learning"[tiab] OR "affective computing"[tiab] OR "cognitive computing"[tiab] OR "knowledge engineering"[tiab] OR "knowledge representation"[tiab] OR "semantic networks"[tiab] OR "reinforcement learning"[tiab] OR "inductive logic programming"[tiab] OR "unsupervised learning"[tiab] OR "pattern recognition"[tiab] OR "feature extraction"[tiab] OR "image analysis"[tiab] OR "text analysis"[tiab] OR "expert systems"[tiab] OR "robot*"[tiab] OR "knowbot*"[tiab] OR "data processing"[tiab] OR "supervised learning"[tiab] OR "semi-supervised learning"[tiab] OR "predictive model"[tiab] OR "virtual agent"[tiab] OR "chatbot"[tiab] OR ("classifier"[tiab] AND ("logistic regression"[tiab] OR "Naïve Bayes"[tiab] OR "Decision trees"[tiab]) OR "virtual reality"[tiab] OR "computer vision"[tiab] OR "constraint satisfaction"[tiab] OR "constraint optimization"[tiab] OR "game theory"[tiab] OR "human computation"[tiab] OR "knowledge-based agent"[tiab]) AND ("child"[mesh] OR "adolescent"[mesh] OR "students"[mesh] OR "pediatrics"[mesh] OR "infant"[mesh] OR "child*"[tiab] OR "adolesc*"[tiab] OR "youth*"[tiab] OR "student*"[tiab] OR "teen*"[tiab] OR "young adult"[tiab] OR "young adults"[tiab] OR "paediatric*"[tiab] OR "pediatric*"[tiab] OR "toddler*"[tiab] OR "infant*"[tiab] OR "caregivers"[mesh] OR "parents"[mesh] OR "family"[mesh] OR "parenting"[mesh] OR "caregiv*"[tiab]

Review	
	OR "parent*"[tiab] OR "family"[tiab] OR "families"[tiab]) AND ("disabled children"[mesh] OR "disab*"[tiab] OR "special needs"[tiab] OR "special need"[tiab] OR "handicap*"[tiab] OR "impair*"[tiab]) AND ("social participation"[mesh] OR "community participation"[mesh] OR "participation"[tiab] OR "engag*"[tiab] OR "attendance"[tiab] OR "involvement"[tiab] OR "inclus*"[tiab])
	("algorithms"[mesh] OR "data mining"[mesh] OR "artificial intelligence"[tiab] OR "machine learning"[tiab] OR "natural language processing"[tiab] OR "data mining"[tiab] OR "algorithm*"[tiab] OR "fuzzy logic"[tiab] OR "knowledge bases"[tiab] OR "neural networks"[tiab] OR "deep learning"[tiab] OR "affective computing"[tiab] OR "cognitive computing"[tiab] OR "knowledge engineering"[tiab] OR "knowledge representation"[tiab] OR "semantic networks"[tiab] OR "reinforcement learning"[tiab] OR "inductive logic programming"[tiab] OR "unsupervised learning"[tiab] OR "pattern recognition"[tiab] OR "feature extraction"[tiab] OR "image analysis"[tiab] OR "text analysis"[tiab] OR "expert systems"[tiab] OR "robot*"[tiab] OR "knowbot*"[tiab] OR "data processing"[tiab] OR "supervised learning"[tiab] OR "semi-supervised learning"[tiab] OR "predictive model"[tiab] OR "virtual agent"[tiab] OR "chatbot" OR ("classifier"[tiab] AND ("logistic regression"[tiab] OR "Naïve Bayes"[tiab] OR "Decision trees"[tiab])) OR "virtual reality"[tiab] OR "computer vision"[tiab] OR "constraint satisfaction"[tiab] OR "constraint optimization"[tiab] OR "game theory"[tiab] OR "human computation"[tiab] OR "knowledge-based agent"[tiab]) AND ("child"[mesh] OR "adolescent"[mesh] OR "students"[mesh] OR "pediatrics"[mesh] OR "infant"[mesh]

	OR "child*"[tiab] OR "adolesc*"[tiab] OR "youth*"[tiab] OR "student*"[tiab] OR "teen*"[tiab] OR "young adult"[tiab] OR "young adults"[tiab] OR "paediatric*"[tiab] OR "pediatric*"[tiab] OR "toddler*"[tiab] OR "infant*"[tiab] OR "caregivers"[mesh] OR "parents"[mesh] OR "family"[mesh] OR "parenting"[mesh] OR "caregiv*"[tiab] OR "parent*"[tiab] OR "family"[tiab] OR "families"[tiab]) AND ("social participation"[mesh] OR "community participation"[mesh] OR "participation"[tiab] OR "engag*"[tiab] OR "attendance"[tiab] OR "involvement"[tiab] OR "inclus*"[tiab]) AND ("rehabilitation"[mesh] OR "health care"[tiab] OR "healthcare"[tiab])
PsychINFO	(MAINSUBJECT.EXACT("artificial intelligence") OR MAINSUBJECT.EXACT("data mining") OR MAINSUBJECT.EXACT("algorithms") OR MAINSUBJECT.EXACT("fuzzy logic") OR AB,TI("artificial intelligence") OR AB,TI("machine learning") OR AB,TI("natural language processing") OR AB,TI("data mining") OR AB,TI("algorithm*") OR AB,TI("fuzzy logic") OR AB,TI("knowledge bases") OR AB,TI("neural networks") OR AB,TI("deep learning") OR AB,TI("affective computing") OR AB,TI("cognitive computing") OR AB,TI("knowledge engineering") OR AB,TI("knowledge representation") OR AB,TI("semantic networks") OR AB,TI("reinforcement learning") OR AB,TI("inductive logic programming") OR AB,TI("unsupervised learning") OR AB,TI("supervised learning") OR AB,TI("semi-supervised learning") OR AB,TI("pattern recognition") OR AB,TI("feature extraction") OR AB,TI("image analysis") OR AB,TI("text analysis") OR AB,TI("expert systems") OR AB,TI("robot*") OR AB,TI("knowbot*") OR AB,TI("data processing") OR AB,TI("predictive model") OR AB,TI("virtual agent") OR AB,TI("chatbot") OR

AB,TI("virtual reality") OR AB,TI("computer vision") OR AB,TI("constraint satisfaction") OR AB,TI("constraint optimization") OR AB,TI("game theory") OR AB,TI("human computation") OR AB,TI("knowledge-based agent") OR(AB,TI("classifier") AND (AB,TI("logistic regression") OR AB,TI("Naïve Bayes") OR AB,TI("Decision trees")))) AND (MAINSUBJECT.EXACT("pediatrics") OR MAINSUBJECT.EXACT("adolescent health") OR MAINSUBJECT.EXACT("child characteristics") OR MAINSUBJECT.EXACT("elementary school student") OR MAINSUBJECT.EXACT("Kindergarten students") OR MAINSUBJECT.EXACT("preschool students") OR AB,TI("child*") OR AB,TI("adolesc*") OR AB,TI("youth*") OR AB,TI("student*") OR AB,TI("teen*") OR AB,TI("young adult") OR AB,TI("young adults") OR AB,TI("paediatric*") OR AB,TI("pediatric*") OR AB,TI("toddler*") OR AB,TI("infant*") OR MAINSUBJECT.EXACT("caregivers") OR MAINSUBJECT.EXACT("family") OR MAINSUBJECT.EXACT("parenting") OR AB,TI("caregiv*") OR AB,TI("parent*") OR AB,TI("family") OR AB,TI("families")) AND (MAINSUBJECT.EXACT("special education students") OR MAINSUBJECT.EXACT("Disorders") OR MAINSUBJECT.EXACT("special needs") OR AB,TI("disab*") OR AB,TI("special needs") OR AB,TI("special need") OR AB,TI("handicap*") OR AB,TI("impair*") OR AB,TI("disorder*")) AND (MAINSUBJECT.EXACT("participation") OR MAINSUBJECT.EXACT("community involvement") OR MAINSUBJECT.EXACT("psychological engagement") OR MAINSUBJECT.EXACT("Activity Theory") OR MAINSUBJECT.EXACT("student engagement") OR MAINSUBJECT.EXACT("mainstreaming (Education)") OR

Review	
	AB,TI("participation") OR AB,TI("engag*") OR AB,TI("attendance") OR AB,TI("involvement") OR AB,TI("inclus*"))
	(MAINSUBJECT.EXACT("artificial intelligence") OR MAINSUBJECT.EXACT("data mining") OR MAINSUBJECT.EXACT("algorithms") OR MAINSUBJECT.EXACT("fuzzy logic") OR AB,TI("artificial intelligence") OR AB,TI("machine learning") OR AB,TI("natural language processing") OR AB,TI("data mining") OR AB,TI("algorithm*") OR AB,TI("fuzzy logic") OR AB,TI("knowledge bases") OR AB,TI("neural networks") OR AB,TI("deep learning") OR AB,TI("affective computing") OR AB,TI("cognitive computing") OR AB,TI("knowledge engineering") OR AB,TI("knowledge representation") OR AB,TI("semantic networks") OR AB,TI("reinforcement learning") OR AB,TI("inductive logic programming") OR AB,TI("unsupervised learning") OR AB,TI("pattern recognition") OR AB,TI("feature extraction") OR AB,TI("image analysis") OR AB,TI("text analysis") AB,TI("supervised learning") OR AB,TI("semi-supervised learning") OR AB,TI("expert systems") OR AB,TI("robot*") OR AB,TI("knowbot*") OR AB,TI("data processing") OR AB,TI("predictive model") OR AB,TI("virtual agent") OR AB,TI("chatbot") OR AB,TI("virtual reality") OR AB,TI("computer vision") OR AB,TI("constraint satisfaction") OR AB,TI("constraint optimization") OR AB,TI("game theory") OR AB,TI("human computation") OR AB,TI("knowledge-based agent") OR (AB,TI("classifier") AND (AB,TI("logistic regression") OR AB,TI("decision trees") OR AB,TI("naïve bayes")))) AND (MAINSUBJECT.EXACT("pediatrics") OR MAINSUBJECT.EXACT("adolescent health") OR MAINSUBJECT.EXACT("child

	characteristics") OR MAINSUBJECT.EXACT("elementary school student") OR MAINSUBJECT.EXACT("Kindergarten students") OR MAINSUBJECT.EXACT("preschool students") OR AB,TI("child*") OR AB,TI("adolesc*") OR AB,TI("youth*") OR AB,TI("student*") OR AB,TI("teen*") OR AB,TI("young adult") OR AB,TI("young adults") OR AB,TI("paediatric*") OR AB,TI("pediatric*") OR AB,TI("toddler*") OR AB,TI("infant*") OR MAINSUBJECT.EXACT("caregivers") OR MAINSUBJECT.EXACT("family") OR MAINSUBJECT.EXACT("parenting") OR AB,TI("caregiv*") OR AB,TI("parent*") OR AB,TI("family") OR AB,TI("families")) AND (MAINSUBJECT.EXACT("participation") OR MAINSUBJECT.EXACT("community involvement") OR MAINSUBJECT.EXACT("psychological engagement") OR MAINSUBJECT.EXACT("Activity Theory") OR MAINSUBJECT.EXACT("student engagement") OR MAINSUBJECT.EXACT("mainstreaming (Education)") OR AB,TI("participation") OR AB,TI("engag*") OR AB,TI("attendance") OR AB,TI("involvement") OR AB,TI("inclus*")) AND (MAINSUBJECT.EXACT("Rehabilitation") OR AB,TI("healthcare") OR AB,TI("health care"))
ERIC	((DE "artificial intelligence") OR (DE "natural learning processing") OR (DE "robotics") OR (DE "Knowledge representation") OR (DE "mathematics") OR (DE "data processing") OR (DE "pattern recognition") OR AB artificial intelligence OR TI artificial intelligence OR AB machine learning OR TI machine learning OR AB natural language processing OR TI natural language processing OR AB data mining OR TI

data mining OR AB algorithm* OR TI algorithm* OR AB fuzzy logic OR TI fuzzy logic OR AB knowledge bases OR TI knowledge bases OR AB neural networks OR TI neural networks OR AB deep learning OR TI deep learning OR AB affective computing OR TI affective computing OR AB cognitive computing OR TI cognitive computing OR AB knowledge engineering OR TI knowledge engineering OR AB knowledge representation OR TI knowledge representation OR AB semantic networks OR TI semantic networks OR AB reinforcement learning OR TI reinforcement learning OR AB inductive logic programming OR TI inductive logic programming OR AB unsupervised learning OR TI unsupervised learning OR AB pattern recognition OR TI pattern recognition OR AB feature extraction OR TI feature extraction OR AB image analysis OR TI image analysis OR AB text analysis OR TI text analysis OR AB expert systems OR TI expert systems OR AB robot* OR TI robot* OR AB knowbot* OR TI knowbot* OR AB data processing OR TI data processing OR AB virtual reality OR TI virtual reality OR AB computer vision OR TI computer vision OR AB constraint satisfaction OR TI constraint satisfaction OR AB constraint optimization OR TI constraint optimization OR AB game theory OR TI game theory OR AB human computation OR TI human computation OR AB knowledge-based agent OR TI knowledge-based agent OR AB supervised learning OR TI supervised learning OR AB semi-supervised learning OR TI semi-supervised learning OR AB predictive model OR TI predictive model OR AB virtual agent OR TI virtual agent OR AB chatbot OR TI chatbot OR (AB classifier OR TI classifier AND (AB logistic regression OR TI logistic regression OR AB naïve bayes OR TI naïve bayes OR AB decision trees OR TI decision trees))) AND ((DE "children") OR (DE

	"adolescents") OR (DE "pediatrics") OR (DE "young adults") OR AB child* OR TI child* OR AB adolesc* OR TI adolesc* OR AB youth* OR TI youth* OR AB student* OR TI student* OR AB teen* OR TI teen* OR AB young adult OR TI young adult OR AB young adults OR TI young adults OR AB paediatric* OR TI paediatric* OR AB pediatric* OR TI pediatric* OR AB toddler* OR TI toddler* OR AB infant* OR TI infant* OR (DE "Family (Sociological Unit)") OR (DE "Caregivers") OR (DE "Parents") OR AB family OR TI family OR AB families OR TI families OR AB caregiv* OR TI caregiv* OR AB parent* OR TI parent*) AND ((DE "student participation") OR (DE "community involvement") OR AB participation OR TI participation OR AB engag* OR TI engag* OR AB attendance OR TI attendance OR AB involvement OR TI involvement OR AB inclus* OR TI inclus*)
	((DE "artificial intelligence") OR (DE "natural learning processing") OR (DE "robotics") OR (DE "Knowledge representation") OR (DE "mathematics") OR (DE "data processing") OR (DE "pattern recognition") OR AB artificial intelligence OR TI artificial intelligence OR AB machine learning OR TI machine learning OR AB natural language processing OR TI natural language processing OR AB data mining OR TI data mining OR AB algorithm* OR TI algorithm* OR AB fuzzy logic OR TI fuzzy logic OR AB knowledge bases OR TI knowledge bases OR AB neural networks OR TI neural networks OR AB deep learning OR TI deep learning OR AB affective computing OR TI affective computing OR AB cognitive computing OR TI cognitive computing OR AB knowledge engineering OR TI knowledge engineering OR AB knowledge representation OR TI knowledge representation OR AB semantic

networks OR TI semantic networks OR AB reinforcement learning OR TI reinforcement learning OR AB inductive logic programming OR TI inductive logic programming OR AB unsupervised learning OR TI unsupervised learning OR AB pattern recognition OR TI pattern recognition OR AB feature extraction OR TI feature extraction OR AB image analysis OR TI image analysis OR AB text analysis OR TI text analysis OR AB expert systems OR TI expert systems OR AB robot* OR TI robot* OR AB knowbot* OR TI knowbot* OR AB data processing OR TI data processing OR AB virtual reality OR TI virtual reality OR AB computer vision OR TI computer vision OR AB constraint satisfaction OR TI constraint satisfaction OR AB constraint optimization OR TI constraint optimization OR AB game theory OR TI game theory OR AB human computation OR TI human computation OR AB knowledge-based agent OR TI knowledge-based agent OR AB supervised learning OR TI supervised learning OR AB semi-supervised learning OR TI semi-supervised learning OR AB predictive model OR TI predictive model OR AB virtual agent OR TI virtual agent OR AB chatbot OR TI chatbot OR (AB classifier OR TI classifier AND (AB logistic regression OR TI logistic regression OR AB naïve bayes OR TI naïve bayes OR AB decision trees OR TI decision trees))) AND ((DE "children") OR (DE "adolescents") OR (DE "pediatrics") OR (DE "young adults") OR AB child* OR TI child* OR AB adolesc* OR TI adolesc* OR AB youth* OR TI youth* OR AB student* OR TI student* OR AB teen* OR TI teen* OR AB young adult OR TI young adult OR AB young adults OR TI young adults OR AB paediatric* OR TI paediatric* OR AB pediatric* OR TI pediatric* OR AB toddler* OR TI toddler* OR AB infant* OR TI infant* OR (DE "Family (Sociological Unit)") OR (DE "Caregivers") OR (DE

	"Parents") OR AB family OR TI family OR AB families OR TI families OR AB caregiv* OR TI caregiv* OR AB parent* OR TI parent*) AND ((DE "student participation") OR (DE "community involvement") OR AB participation OR TI participation OR AB engag* OR TI engag* OR AB attendance OR TI attendance OR AB involvement OR TI involvement OR AB inclus* OR TI inclus*) AND ((DE "rehabilitation") OR (DE "health services") OR AB rehabilitation OR TI rehabilitation OR AB health care OR TI health care OR AB healthcare OR TI healthcare)
CINAHL	((MH "artificial intelligence+") OR (MH "algorithms+") OR (MH "data mining+") OR AB("artificial intelligence") OR TI("artificial intelligence") OR AB("machine learning") OR TI("machine learning") OR AB("natural language processing") OR TI("natural language processing") OR AB("data mining") OR TI("data mining") OR AB("algorithm*") OR TI("algorithm*") OR AB("fuzzy logic") OR TI("fuzzy logic") OR AB("knowledge bases") OR TI("knowledge bases") OR AB("neural networks") OR TI("neural networks") OR AB("deep learning") OR TI("deep learning") OR AB("affective computing") OR TI("affective computing") OR AB("cognitive computing") OR TI("cognitive computing") OR AB("knowledge engineering") OR TI("knowledge engineering") OR AB("knowledge representation") OR TI("knowledge representation") OR AB("semantic networks") OR TI("semantic networks") OR AB("reinforcement learning") OR TI("reinforcement learning") OR AB("inductive logic programming") OR TI("inductive logic programming") OR AB("unsupervised learning") OR TI("unsupervised learning") OR AB("pattern recognition") OR TI("pattern recognition") OR AB("feature extraction") OR

TI("feature extraction") OR AB("image analysis") OR TI("image analysis") OR AB("text analysis") OR TI("text analysis") OR AB("expert systems") OR TI("expert systems") OR AB("robot*") OR TI("robot*") OR AB("knowbot*") OR TI("knowbot*") AB("supervised learning") OR TI("supervised learning") OR AB("semi-supervised learning") OR TI("semi-supervised learning") OR AB("predictive model") OR TI("predictive model") OR AB("virtual agent") OR TI("virtual agent") OR AB("chatbot") OR TI("chatbot") OR TI("virtual reality") OR AB("virtual reality") OR TI("computer vision") OR AB("computer vision") OR TI("constraint satisfaction") OR AB("constraint satisfaction") OR TI("constraint optimization") OR AB("constraint optimization") OR TI("game theory") OR AB("game theory") OR TI("human computation") OR AB("human computation") OR TI("knowledge-based agent") OR AB("knowledge-based agent") OR (TI("classifier") OR AB("classifier") AND (TI("logistic regression") OR AB("logistic regression") OR TI("decision trees") OR AB("decision trees") OR TI("naïve bayes") OR AB("naïve bayes")))) AND ((MH "child+") OR (MH "adolescence+") OR (MH "students+") OR (MH "pediatrics+") OR AB("child*") OR TI("child*") OR AB("adolesc*") OR TI("adolesc*") OR AB("youth*") OR TI("youth*") OR AB("student*") OR TI("student*") OR AB("teen*") OR TI("teen*") OR AB("young adult") OR TI("young adult") OR AB("young adults") OR TI("young adults") OR AB("paediatric*") OR TI("paediatric*") OR AB("pediatric*") OR TI("pediatric*") OR AB("toddler*") OR TI("toddler*") OR AB("infant*") OR TI("infant*") OR (MH "caregivers+") OR (MH "parents of disabled children+") OR (MH "family+") OR AB("caregiv*") OR TI("caregiv*") OR AB("parent*") OR TI("parent*") OR AB("family") OR TI("family") OR AB("families") OR TI("families"))

	AND (AB("disab*") OR TI("disab*") OR AB("special needs") OR TI("special needs") OR AB("special need") OR TI("special need") OR AB("handicap*") OR TI("handicap*") OR AB("impair*") OR TI("impair*")) AND ((MH "social participation+") OR (MH "leisure participation+") OR (MH "play participation+") OR (MH "social involvement+") OR AB("participation") OR TI("participation") OR AB("engag*") OR TI("engag*") OR AB("attendance") OR TI("attendance") OR AB("involvement") OR TI("involvement") OR AB("inclus*") OR TI("inclus*"))
	((MH "artificial intelligence+") OR (MH "algorithms+") OR (MH "data mining+") OR AB("artificial intelligence") OR TI("artificial intelligence") OR AB("machine learning") OR TI("machine learning") OR AB("natural language processing") OR TI("natural language processing") OR AB("data mining") OR TI("data mining") OR AB("algorithm*") OR TI("algorithm*") OR TI("fuzzy logic") OR AB("knowledge bases") OR TI("knowledge bases") OR AB("neural networks") OR TI("neural networks") OR AB("deep learning") OR TI("deep learning") OR AB("affective computing") OR TI("affective computing") OR AB("cognitive computing") OR TI("cognitive computing") OR AB("knowledge engineering") OR TI("knowledge engineering") OR AB("knowledge representation") OR TI("knowledge representation") OR AB("semantic networks") OR TI("semantic networks") OR AB("reinforcement learning") OR TI("reinforcement learning") OR AB("inductive logic programming") OR TI("inductive logic programming") OR AB("unsupervised learning") OR TI("unsupervised learning") OR AB("pattern recognition") OR TI("pattern recognition") OR AB("feature extraction") OR TI("feature extraction")

OR AB("image analysis") OR TI("image analysis") OR AB("text analysis") OR TI("text analysis") OR AB("expert systems") OR TI("expert systems") OR AB("robot*") OR TI("robot*") OR AB("knowbot*") OR TI("knowbot*") OR AB("supervised learning") OR TI("supervised learning") OR AB("semi-supervised learning") OR TI("semi-supervised learning") OR AB("predictive model") OR TI("predictive model") OR AB("virtual agent") OR TI("virtual agent") OR AB("chatbot") OR TI("virtual reality") OR AB("virtual reality") OR TI("computer vision") OR AB("computer vision") OR TI("constraint satisfaction") OR AB("constraint satisfaction") OR TI("constraint optimization") OR AB("constraint optimization") OR TI("game theory") OR AB("game theory") OR TI("human computation") OR AB("human computation") OR TI("knowledge-based agent") OR AB("knowledge-based agent") OR TI("chatbot") OR (TI("classifier") OR AB("classifier") AND (TI("logistic regression") OR AB("logistic regression") OR TI("decision trees") OR AB("decision trees") OR TI("naïve bayes") OR AB("naïve bayes")))) AND ((MH "child+") OR (MH "adolescence+") OR (MH "students+") OR (MH "pediatrics+") OR AB("child*") OR TI("child*") OR AB("adolesc*") OR TI("adolesc*") OR AB("youth*") OR TI("youth*") OR AB("student*") OR TI("student*") OR AB("teen*") OR TI("teen*") OR AB("young adult") OR TI("young adult") OR AB("young adults") OR TI("young adults") OR AB("paediatric*") OR TI("paediatric*") OR AB("pediatric*") OR TI("pediatric*") OR AB("toddler*") OR TI("toddler*") OR AB("infant*") OR TI("infant*") OR (MH "caregivers+") OR (MH "parents of disabled children+") OR (MH "family+") OR AB("caregiv*") OR TI("caregiv*") OR AB("parent*") OR TI("parent*") OR AB("family") OR TI("family") OR AB("families") OR TI("families")) AND ((MH "social

	participation+") OR (MH "leisure participation+") OR (MH "play participation+") OR (MH "social involvement+") OR AB("participation") OR TI("participation") OR AB("engag*") OR TI("engag*") OR AB("attendance") OR TI("attendance") OR AB("involvement") OR TI("involvement") OR AB("inclus*") OR TI("inclus*")) AND ((MH "rehabilitation+") OR (MH "Health care delivery+") OR AB("rehabilitation") OR TI("rehabilitation") OR AB("healthcare") OR TI("healthcare") OR AB("health care") OR TI("health care"))
IEEE Xplore	("Abstract":"artificial intelligence" OR "Abstract":"machine learning" OR "Abstract":"natural language processing" OR "Abstract":"data mining" OR "Abstract":algorithm OR "Abstract":"fuzzy logic" OR "Abstract":"knowledge bases" OR "Abstract":"neural networks" OR "Abstract":"deep learning" OR "Abstract":"affective computing" OR "Abstract":"cognitive computing" OR "Abstract":"knowledge engineering" OR "Abstract":"knowledge representation" OR "Abstract":"semantic networks" OR "Abstract":"reinforcement learning" OR "Abstract":"inductive logic programming" OR "Abstract":"unsupervised learning" OR "Abstract":"pattern recognition" OR "Abstract":"feature extraction" OR "Abstract":"image analysis" OR "Abstract":"text analysis" OR "Abstract":"expert systems" OR "Abstract":robot OR "Abstract":knowbot OR "Abstract":"data processing" OR "Abstract":"supervised learning" OR "Abstract":"semi-supervised learning" OR "Abstract":"predictive model" OR "Abstract":"virtual agent" OR "Abstract":"chatbot" OR "Abstract":"virtual reality" OR "Abstract":"computer vision" OR "Abstract":"constraint satisfaction" OR "Abstract":"constraint optimization" OR

	"Abstract":"game theory" OR "Abstract":"human computation" OR "Abstract":"knowledge-based agent" OR "Abstract":classifier AND ("logistic regression" OR "decision trees" OR "naïve bayes")) AND ("Abstract":disab* OR "Abstract":"special needs" OR "Abstract":"special need" OR "Abstract":handicap* OR "Abstract":impair* OR "Abstract":disorder) AND ("Abstract":child OR "Abstract":children OR "Abstract":adolescent OR "Abstract":adolescence OR "Abstract":youth OR "Abstract":student OR "Abstract":teen* OR "Abstract":"young adult" OR "Abstract":paediatric OR "Abstract":pediatric OR "Abstract":toddler OR "Abstract":infant OR "Abstract":caregiver OR "Abstract":parent OR "Abstract":family OR "Abstract":families) AND ("Abstract":participation OR "Abstract":engagement OR "Abstract":attendance OR "Abstract":involvement OR "Abstract":inclusion OR "Abstract":inclusive)
	(("Abstract":"classifier" AND ("Abstract":"logistic regression" OR "Abstract":"decision trees" OR "Abstract":"naïve bayes")) OR "Abstract":"artificial intelligence" OR "Abstract":"machine learning" OR "Abstract":"natural language processing" OR "Abstract":"data mining" OR "Abstract":algorithm OR "Abstract":"fuzzy logic" OR "Abstract":"knowledge bases" OR "Abstract":"neural networks" OR "Abstract":"deep learning" OR "Abstract":"affective computing" OR "Abstract":"cognitive computing" OR "Abstract":"knowledge engineering" OR "Abstract":"knowledge representation" OR "Abstract":"semantic networks" OR "Abstract":"reinforcement learning" OR "Abstract":"inductive logic programming" OR "Abstract":"unsupervised learning" OR "Abstract":"pattern recognition" OR "Abstract":"feature extraction" OR "Abstract":"image analysis" OR "Abstract":"text

	analysis" OR "Abstract":"expert systems" OR "Abstract":robot OR "Abstract":knowbot OR "Abstract":"data processing" OR "Abstract":"supervised learning" OR "Abstract":"semi-supervised learning" OR "Abstract":"predictive model" OR "Abstract":"virtual agent" OR "Abstract":chatbot OR "Abstract":"virtual reality" OR "Abstract":"computer vision" OR "Abstract":"constraint satisfaction" OR "Abstract":"constraint optimization" OR "Abstract":"game theory" OR "Abstract":"human computation" OR "Abstract":"knowledge-based agent") AND ("Abstract":rehabilitation OR "Abstract":therap* OR "Abstract":"health care" OR "Abstract":healthcare) AND ("Abstract":child OR "Abstract":children OR "Abstract":adolescent OR "Abstract":adolescence OR "Abstract":youth OR "Abstract":student OR "Abstract":teen* OR "Abstract":"young adult" OR "Abstract":paediatric OR "Abstract":pediatric OR "Abstract":toddler OR "Abstract":infant OR "Abstract":caregiver OR "Abstract":parent OR "Abstract":family OR "Abstract":families) AND ("Abstract":participation OR "Abstract":engagement OR "Abstract":attendance OR "Abstract":involvement OR "Abstract":inclusion OR "Abstract":inclusive)
ACM Digital Library	("artificial intelligence" OR "machine learning" OR "natural language processing" OR "data mining" OR algorithm* OR "fuzzy logic" OR "knowledge bases" OR "neural networks" OR "deep learning" OR "affective computing" OR "cognitive computing" OR "knowledge engineering" OR "knowledge representation" OR "semantic networks" OR "reinforcement learning" OR "inductive logic programming" OR "unsupervised learning" OR "pattern recognition" OR "feature extraction" OR "image analysis" OR "text analysis" OR "expert systems" OR robot* OR knowbot* OR

	"data processing" OR "supervised learning" OR "semi-supervised learning" OR "predictive model" OR "virtual agent" OR "chatbot" OR "virtual reality" OR "computer vision" OR "constraint satisfaction" OR "constraint optimization" OR "game theory" OR "human computation" OR "knowledge-based agent" OR ("classifier" AND ("logistic regression" OR "decision trees" OR "naïve bayes"))) AND (participation OR engag* OR attendance OR involvement OR inclus*) AND (disab* OR "special needs" OR "special need" OR handicap* OR impair* OR disorder*) AND (child* OR adolesc* OR youth* OR student* OR teen* OR "young adult" OR "young adults" OR paediatric* OR pediatric* OR toddler* OR infant* OR caregiv* OR parent* OR family OR families)
	("artificial intelligence" OR "machine learning" OR "natural language processing" OR "data mining" OR algorithm* OR "fuzzy logic" OR "knowledge bases" OR "neural networks" OR "deep learning" OR "affective computing" OR "cognitive computing" OR "knowledge engineering" OR "knowledge representation" OR "semantic networks" OR "reinforcement learning" OR "inductive logic programming" OR "unsupervised learning" OR "pattern recognition" OR "feature extraction" OR "image analysis" OR "text analysis" OR "expert systems" OR robot* OR knowbot* OR "data processing" OR "supervised learning" OR "semi-supervised learning" OR "predictive model" OR "virtual agent" OR "chatbot" OR "virtual reality" OR "computer vision" OR "constraint satisfaction" OR "constraint optimization" OR "game theory" OR "human computation" OR "knowledge-based agent" OR ("classifier" AND ("logistic regression" OR "decision trees" OR "naïve bayes"))) AND

**Review**	(participation OR engag* OR attendance OR involvement OR inclus*) AND (child* OR adolesc* OR youth* OR student* OR teen* OR "young adult" OR "young adults" OR paediatric* OR pediatric* OR toddler* OR infant* OR caregiv* OR parent* OR family OR families) AND (rehabilitation OR therap* OR "health care" OR healthcare)
