# Supplementary material for: Artificial Intelligence in Rehabilitation Targeting the Participation of Children and Youth With Disabilities: Scoping Review
Source: J Med Internet Res. 2021 Nov 4;23(11):e25745. doi: 10.2196/25745 (PMC8603165; doi:10.2196/25745)
Supplement: Multimedia Appendix 2 [file jmir_v23i11e25745_app2.pdf]

Review

Appendix B. Included studies.

Study	Sample (n)	Child/youth age, Mean(SD); range [years]	Child/youth gender, male (%)	Child/youth diagnosis§	Race/ethnicity, socioeconomic status, parental education, or family income
Adams et al. [52]	1 child	12	0	CP	NR
Alotaibi et al. [81]	5 children	Mean(SD): 7.8(3.1); range: 4-12	80	Hemiplegia, Diplegia	NR
Alnajjar et al. [86]	11 children	Mean(SD): 9.0(2.6)	100	ASD	NR
Altizer et al. [121]	15 children, 6 caregivers	NR	NR	ASD	NR
Arman et al. [112]	50 children	Mean(SD): 12.8	16	Juvenile idiopathic arthritis	NR
Arpaia et al. [155]	4 children	range: 6-8	NR	ADHD	NR
Aslam et al. [50]	4 students	Mean(SD): 13.8(2.6); range: 3-19	75	ID	NR

Barakova et al. [78]	6 children	Mean(SD): 10.3(1.6); range: 8-12	100	ASD	NR
Barnes et al. [87]	15 children	NR	NR	ASD, TD	NR
Beaudry et al. [128]	13 youth	Mean(SD): 15.7(0.9); range: 14-17	NR	Mixed	NR
Bers et al. [108]	7 children	Range: 7-18	NR	End-stage renal disease	NR
Besio et al. [49]	7 children	Mean(SD): 9.6(1.9); range: 7-22	86	CP, degenerative muscle disease	NR
Bian et al. [107]	20 children	Mean(SD): 15.5	90	ASD	NR
Black et al. [126]	3 children	Mean(SD): 14.4(2.0); range: 12-16	33	CP	NR
Blanson Henkemans et al. [76]	27 children	Mean(SD): 11.0(1.7)	48	Diabetes	NR

Blanson Henkemans et al. [77]	5 children	Mean(SD): 10.2(1.3); range: 9-12	60	Diabetes	NR
Boccanfuso et al. [75]	4 children	NR	NR	ASD, TD	NR
Bonarini et al. [48]	11 children, 2 therapists, 3 specialists	Range: 3-10	NR	ASD, DS, ID, Prader-Willy syndrome, psychosis	NR
Bonney et al. [115]	43 children	Mean (SD): 14.3(1.1); range: 13-16	0	DCD	NR
Bortone et al. [123]	8 children	Mean(SD): 9.75	75	CP, developmental dyspraxia	NR
Buitrago et al. [74]	1 child	8	100	CP	NR
Bulgarelli et al. [84]	6 children	Mean(SD): 9.2(1.5); range: 7-11	83	CP	NR
Butchart et al. [104]	5 children, 5 mothers	Mean(SD): 10.0(2.35); range: 6-12	60	CP, hemispherectomy,	NR

				hereditary spastic paresis	
Byrne et al. [124]	72 children	Mean(SD): 13.2; range: 6-20	NR	ID	NR
Conchinha et al. [46]	3 children	Mean(SD): 14.7(0.6); range: 14-15	75	Learning disability	NR
Conn Welch et al. [53]	26 children	Mean(SD): 15.8; range: 13-18	77	ASD, TD	NR
Cook et al. [45]	12 children	Mean(SD): 9.9; range: 6-14	NR	PD	NR
Cook et al. [73]	4 children	Range: 6-7	NR	CP	NR
Costa el al. [94]	2 youth	NR	NR	ASD	NR
Encarnação et al. [72]	9 children, 9 regular teachers, 9 special	Range: 3-6	67	CP, TBI, DD	NR

	education teachers				
Ferm et al. [71]	4 children	Mean(SD): 5.2(0.9); range: 4-7	25	CP, TD	NR
Fels et al. [95]	3 students NR teachers NR parents	Mean(SD): 11(1.41)	33	Chronic Renal Failure	NR
Feng [99]	16 children	Mean(SD): ASD: 11.73(3.11) TD: 10.22(2.06)	88	TD, ASD	NR
Gilliaux et al. [44]	16 children	Mean(SD): 10.9	NR	CP	NR
Goldberg et al. [110]	5 youth	NR	NR	Wheelchair users, VI	NR
Goodrich et al. [43]	2 children	Mean(SD): 5.8(3.2); range: 3-8	NR	ASD	NR
Green et al. [116]	4 children	Mean(SD): 6.25(4.49);	50	hemiplegia	NR

		range: 3-14			
Haumont et al. [70]	3 children/youth	Mean(SD): 14.3(3.5); range: 11-18	66	AMC, SMA type II	NR
Iacono et al. [42]	10 children	Mean(SD): 8.3(0.6)	90	ASD	NR
Ismail et al. [89]	20 children	Mean(SD): 10.85; range: 9-12	75	Cognitive impairment	NR
Javed et al. [40]	17 children	Mean(SD): 7.6; range: 4-12	82	ASD, TD	White, Asian
Javed et al. [41]	18 children	Mean(SD): 15.3; range: 4-11	72	ASD, TD	White, Asian
Jung et al. [68]	1 child	8	100	ASD	Latino-American
Ke et al. [118]	4 children	Mean(SD): 9.75(.43); range: 9-10	50	ASD	Non Hispanic White, Non Hispanic Black

Khamassi et al. [67]	12 children	NR	NR	ASD	NR
Kim et al. [66]	24 children	Mean(SD): 9.4(2.4); range: 4-13	88	ASD	NR
Kim et al [96]	29 children	Mean(SD): 11.3; range: 9-14	83	ASD	NR
Klein et al. [37]	3 children	Range: 3-5	33	CP, DD	NR
Koch et al. [65]	48 children	Mean(SD): 8.5; range: 5-12	52	ASD, TD	Hispanic/Latino, Non-Hispanic White, Non-Hispanic Black, other (mixed)
Kolne et al. [111]	31 children	Range: 6-14	74	ASD, CP, DD, skeletal dysplasia	NR
Kokkoni et al. [64]	3 children	Mean(SD): 1.3(0.5); range: 0.8-2	NR	DS, TD	NR
Kozima et al. [39]	3 children	Mean(SD): 3.0; range: 3-3	33	ASD	NR

Kozima et al. [63]	3 children	Mean(SD): 3.0(1.0); range: 3-3	33	ASD	NR
Lahiri et al. [120]	8 children	Mean(SD): 16.07(2.09); range: 13-18.25	NR	ASD	NR
Lehmann et al. [106]	10 children	Mean(SD): 8.3(0.6)	90	ADHD, ASD, DS	NR
Lindsay et al. [61]	21 children, 21 parents, 11 program staff	Range: 6-8	86	ASD, PD, brain injury	NR
Lindsay et al. [62]	18 children/youth, 12 parents, 11 clinicians/ staff/ volunteers	Range: 9-14	78	Mixed	NR
Ljunglöf et al. [105]	3 children	Mean(SD): 5.0(1.0); range: 4-6	67	CP	NR
Ludi et al. [60]	19 children	NR	89	VI	NR

Ludi et al. [102]	46 children	NR	89	VI, multiple disabilities	Range of socioeconomic groups
Marti et al. [59]	NR	NR	NR	Mixed	NR
Marti et al. [80]	9 children	Range: 6-11	33	Cognitive impairment, developmental disorder, epilepsy, ADHD, Sclerosi tuberosa, Pierre Robin sequence	NR
Marti et al. [91]	3 youth	Mean(SD): 21.3(6.6); range: 14-27	33	DS, Hanhart and Moebius syndrome	NR
Mei et al. [122]	10 youth	NR	NR	ASD	NR
Metatla et al. [88]	7 children, 3 teaching assistants	NR	NR	VI	NR
Miller et al. [114]	19 children	Mean(SD): 10.1; range: 8-13	63	CP	NR

Mineo et al. [117]	42 children	Mean(SD): 9.93(3.45); range: 6-18	NR	ASD	NR
Miyamoto et al. [101]	2 children	Mean(SD): 10(0)	50	ASD	NR
Neto et al. [93]	20 students	Mean(SD): 6.6(1.1)	65	VI, multiple impairments	NR
Newhart et al. [51]	5 children, 5 parents, 10 teachers, 35 classmates, 6 school/ district administrators	Range: 6-16	80	Chronic illness	NR
Newhart et al. [100]	19 children, 16 guardians, 20 teachers, 16 school administrators, 44 classmates	Range: 5-18	53	Cancer, SMA, immunodeficiency disorder, heart failure, injury	NR

Otoom et al. [127]	9 children	Range: 18-22	67	Deaf	NR
Pioggia et al. [69]	4 children	Mean(SD): 11.8(5.6); range: 7-20	75	ASD	NR
Pliasa et al. [57]	12 children, 3 teachers	Mean(SD): 7(1.3); range: 6-9	83	ASD, TD	NR
Porayska-Pomsta et al. [109]	21 children	Mean(SD): 7; range: 4-14	93	ASD, TD	NR
Pulido et al. [56]	120 children	Mean(SD): 7.8	NR	Motor impairment, TD	NR
Reid [119]	13 children	Mean(SD): 10.41(1.25); Range: 8-12	54	CP	NR
Reid [113]	3 children	Mean(SD): 6.3(4.0); range: 2-10	NR	Quadriplegia, diplegia	NR
Ríos-Rincón et al. [38]	4 children, 4 mothers	Mean(SD): 7.5(1.9); range: 5-9	75	CP	NR

Rivas-Perez et al. [125]	3 children	NR	NR	VI	NR
Rudovic et al. [58]	30 children	Range: 3-13	NR	ASD	Asian, Non-Hispanic White
Santatiwongchai et al. [82]	6 children	Range: 3-10	83	ASD	NR
Silva et al. [90]	10 children	Mean(SD): 7; Range: 5-8	100	ASD	NR
Schroeder et al. [83]	18 children/youth	Mean(SD): 11.4(4.9); range: 5-21	78	CP	NR
Shamsuddin et al. [97]	1 child	Mean(SD): 10	100	ASD	NR
Silvera-Tawil et al. [47]	28 children/youth, 6 caregivers, 6 teachers, 6 school staff	Range: 13-19	NR	ASD, ID	Low socio-economic background
Simut et al. [98]	30 children	Mean(SD): 6.67(.92); range: 5-8	90	ASD	NR

So et al. [36]	23 children	Mean(SD): 5.3; range: 4-6	87	ASD	NR
Soares et al. [85]	1 child	NR	NR	Mixed	NR
Stanton et al. [55]	11 children	Range: 5-8	91	ASD	NR
Wainer et al. [54]	6 children	Mean(SD): 6.5(0.8); range: 6-8	83	ASD	NR
Weibel et al. [92]	4 children, 1 teacher	Mean(SD): 13.25; range: 12-14	25	Cancer	NR
Yee et al. [35]	5 children	Mean(SD): 4.8; range: 4-5	80	ASD	NR
Zhanatkyzy et al. [103]	21 children	Range: 4-8	86	ASD	NR

Note: PD=physical disability, DCD= developmental coordination disorder, DS=Down syndrome, ASD=autism spectrum disorder, TD=typically developing, CP=cerebral palsy, ADHD=attention deficit hyperactive disorder, AMC=arthrogryposis multiplex congenita, SMA=spinal muscular atrophy, DD= developmental delay, VI= visual impairment, ID= intellectual disability, NR= not reported

Review

§ Designed and/or tested
